# Building a novel strategic research agenda for METROFOOD-RI: design process and multi-stakeholder engagement towards thematic prioritization

**DOI:** 10.3389/fnut.2023.1151611

**Published:** 2023-06-23

**Authors:** Tamara Stelzl, Maria Z. Tsimidou, Nastasia Belc, Claudia Zoani, Michael Rychlik

**Affiliations:** ^1^Department of Life Science Engineering, Chair of Analytical Food Chemistry, TUM School of Life Sciences, Technical University of Munich, Freising, Germany; ^2^Food Chemistry and Technology Laboratory, School of Chemistry, Aristotle University of Thessaloniki, Thessaloniki, Greece; ^3^National R&D Institute for Food Bioresources, IBA Bucharest, Bucharest, Romania; ^4^Department for Sustainability of Production and Territorial Systems, Biotechnologies and Agro-Industry Division, Casaccia Research Center, ENEA, Italian National Agency for New Technologies, Energy and Sustainable Economic Development, Rome, Italy

**Keywords:** METROFOOD-RI, European research infrastructures, research agenda, food metrology, agrifood, European Strategy Forum on Research Infrastructures (ESFRI)

## Abstract

**Introduction:**

The European research landscape suffers widely from fragmentation and little cross-border research collaboration. Efforts are underway to bring the European Research Area to a higher level of performance and capacity in cutting-edge science, with high anticipations for the promotion of multidisciplinary research infrastructures of transnational engagement. A European distributed research infrastructure active in this framework is METROFOOD-RI, committed to promoting metrology in food and nutrition with particular focus on measurement research related to agrifood systems.

**Methods:**

For research infrastructures, streamlining resources among partner organizations and establishing priorities around specific topics is critical for ensuring smooth operation. Similarly, METROFOOD-RI faced the challenge of exploring its strategic direction and research priorities as revealed in its first Strategic Research and Innovation Agenda (SRIA). This report details how the internal process of topic identification and prioritization progressed within the METROFOOD-RI SRIA and what obstacles were encountered along the way. A dual-track strategy was taken for locating future SRIA topics, applying a top-down and bottom-up approach, followed by internal consultation with METROFOOD-RI experts. The topic prioritization drew on a vote among the METROFOOD-RI Management Committee employing a custom-designed numerical rating scale questionnaire. Based on the maximum scores obtained for each topic, appropriate thresholds were introduced for classifying individual topics into high, medium, low, and very low priority ones.

**Results:**

A total of 80 topics categorized into eight major clusters of challenges were located as potential SRIA candidates. Upon prioritization, 9 topics of very high priority and 16 topics of medium priority were identified as key research thematic areas of the newly developed SRIA.

**Discussion:**

As a strategic framework, the SRIA occupies a central position and sets not only the scientific focus of the research infrastructure in the coming years, but also contributes to realizing the full potential and excellence of METROFOOD-RI, selectively expanding the existing portfolio and thus contributing to maximum efficiency and sustainability. It is anticipated that the lessons learned by METROFOOD-RI and its experiences shared are a valuable stimulus and guide for those who are taking on the challenge of setting-up a SRIA and are looking for edifying and constructive information on how to do so.

## 1. Introduction

European research infrastructures (RIs) have become indispensable tools for fostering global collaboration and partnerships in research and development, accelerating knowledge creation and sharing of technologies and resources across scientific disciplines, and driving scientific innovation and progress along with problem-solving capabilities, countering policy fragmentation, and tackling various societal challenges at hand by closing prevailing knowledge gaps.

METROFOOD-RI^1^—*a pan-European distributed Research Infrastructure (RI) for promoting Metrology in Food and Nutrition*—is one of Europe’s RIs standing at the beginning of its business lifecycle and currently undergoing transformation from the preparatory to an operational stage. Under Italian auspices, METROFOOD-RI is presently made up of research institutions (research centers, national metrology institutes, universities, etc.) from 18 EU-Member States and Associated Countries. It is founded to provide a portfolio of professional integrated physical and electronic services, including access to top-class laboratories, facilities and testing sites, and electronic resources. The goal to drive standardization and harmonization of measurements in support to the agrifood sector (e.g., food quality, composition and labelling, safety, hygiene, authenticity, traceability, sustainability and circular bioeconomy) includes sharing collected food data and integrating existing knowledge, experience and the latest technologies to make them available to various audiences such as food inspection agencies, researchers, policy makers, food business operators, consumers/citizens and for educational purposes. A comprehensive overview of the METROFOOD-RI service offerings has been outlined by Vandermeiren et al. (1). In view of the global scientific research arena, the activities of METROFOOD-RI are primarily aimed at enhancing scientific excellence in the areas of food quality and safety, traceability of raw materials and products, the sustainability of agrifood systems and food transparency, more specifically related to data collection and reliability of food data measurement, as well as to fundamental and cutting-edge scientific research focused on food and nutrition (2). In providing high-quality metrology services in this respect, *inter alia* in support of the digitalization of agrifood systems, METROFOOD-RI represents an important transversal interface along the entire food value chain, bringing together multidisciplinary and interconnected scientific domains down the longitudinal-axis from farm to fork (including primary food production, food manufacturing and processing, storage and distribution, retailing, preparation and home storage, consumption, and waste). Thus, it is pioneering the field over other food-related research infrastructures serving only distinct aspects such as primary production in the food chain spectrum (e.g., EMPHASIS, AnaEE, EU-IBISBA) (3).

The establishment of joint large-scale research infrastructures requires in general the definition of strategic priorities and the formulation of shared goals to enable and efficiently drive coordinated and efficient work as a team towards common objectives. The long-term visions and missions as well as the long-term actions of, for example, research organizations, networks or committees, are usually outlined in proprietary Strategic Research and Innovation Agendas (SRIA), which are commonly based on a prior comprehensive inventory of existing policy needs, research gaps, or complementary opportunities, thus pinpointing relevance as well as existing or untapped innovation potentials.

The operation of European research infrastructures such as METROFOOD-RI also demands adherence to a specific strategy and an operational framework, preferably defined in a SRIA, in line with the guidelines of the European Strategy Forum on Research Infrastructures (ESFRI), as the main strategic body entrusted by the Council of the European Union with the administrative planning, implementation and monitoring of all research infrastructures in Europe (4). Established in 2002, the ESFRI legal entity accommodates delegates from national research ministries of the EU-Member States and associated countries, along with representatives of the European Commission, and is supported by thematic working groups. ESFRI plays a central role in supporting EU policy in the strategic planning and implementation of multi-annual and coherent large-scale research infrastructures, which are set out in the ESFRI Roadmap serving as a high-level strategic work plan. Starting as an emerging ESFRI project in 2016, METROFOOD-RI has successfully entered the ESFRI Roadmap in the “Health & Food Sector” in 2018 (5), was further prepared for operative deployment by mid-2021 (METROFOOD-PP project, GA No. 871083) and is now close to full commissioning under the legal status of a European Research Infrastructure Consortium (ERIC).

The focus of the present article is on a comprehensive account of the conceptualization and design process of a SRIA for METROFOOD-RI. To develop a SRIA that meets the aspirations and mission, capabilities and resources of METROFOOD-RI and realizes its full potential, also in terms of ensuring sustainable operational performance, along with its mandate to provide effective solutions to major societal problems and emerging issues, a focused approach was required. A literature research conducted to gather ex ante information on how the SRIA preparation process leading up to the launch of the final agenda evolved in other research infrastructures or research initiatives (6) for guidance, revealed a wide range of different procedures and workflows, while the vast majority did not disclose any information in this regard. More generally, there is apparently no one-size-fits-all approach when it comes to the elaboration of a coherent and tailor-made SRIA. In practice, the following techniques are widely applied, also in combination: (1) consultations and dialogues with stakeholders/communities and the organization of thematic workshops (7, 8); (2) analysis of trends, (technological/scientific) developments, emerging and existing (research) challenges (8, 9); (3) identification of priority issues in key areas of innovation (9); (4) exploration of (emerging) policy priorities (9, 10); (5) alignment of actions with strategic objectives and (competing) actors or activities (7). Although this list does not claim to be exhaustive, it illustrates that a series of different considerations need to be taken into account on a case-by-case basis when drawing up a SRIA. The various aspects should ideally be weighed up against each other and can sometimes make a SRIA project a rather lengthy, resource-intensive and difficult task. This was also the challenge METROFOOD-RI faced in developing its first SRIA, and the following section describes in more detail how this demanding task was accomplished. In this regard, in-depth insights are presented on the overall SRIA formulation process and the approaches taken to identify and prioritize target areas for action, culminating in a consensus decision on future priority topics to be addressed within the research infrastructure.

## 2. Materials and methods

### 2.1. Strategic approach towards exploring SRIA relevant topics

**Figure 1:**
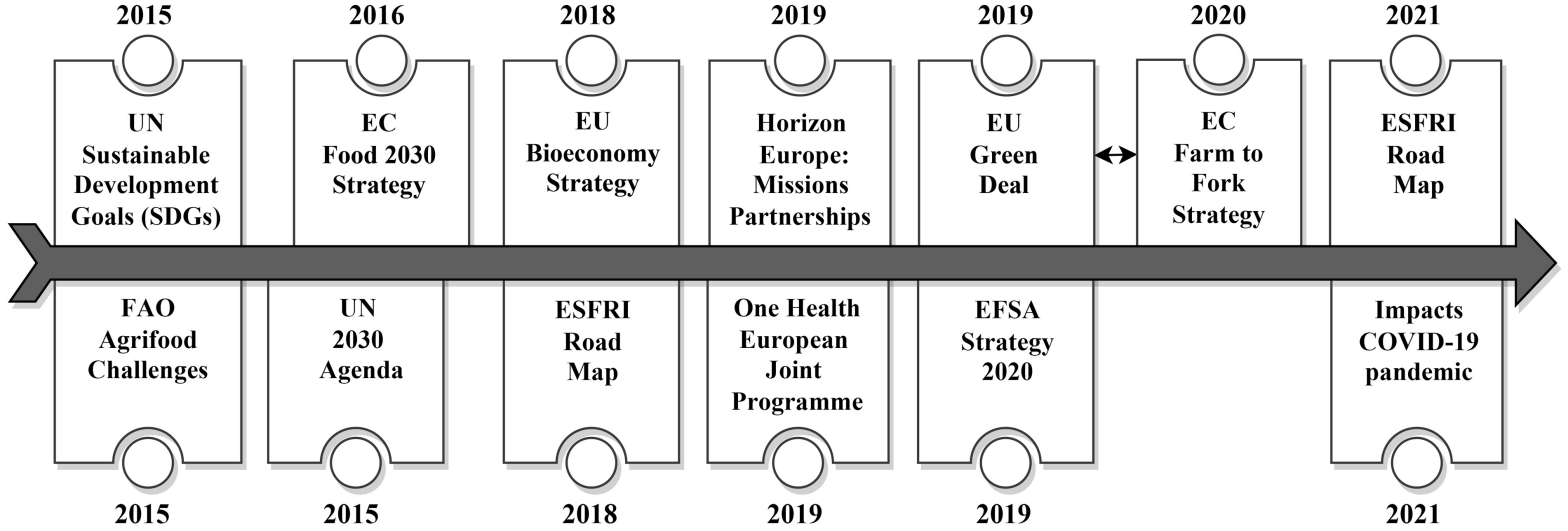
Key strategies and policies linked to the agrifood considered in developing the SRIA for METROFOOD-RI.

The strategy pursued by METROFOOD-RI for drafting its first SRIA was essentially based on two pillars by adopting successively a top-down and bottom-up approach. The bottom-up approach, as detailed in the METROFOOD-RI Scientific Plan published by Tsimidiou et al. (11), relied on a systematic literature review dating back to 2015 using specific keyword searches centered on the broader themes of metrology (in food/feed/environmental matrices and food contact materials analyses), food security, and sustainability of agrifood systems and circular economy. This research study helped to generate deeper insights into current research priorities related to the aforementioned key topics, and to identify new and emerging issues of relevance and concern to public health, as well as priority issues of common interest (to policy makers, researchers, food businesses, etc.). As part of METROFOOD-RI strategic planning efforts, the knowledge gathered on short-and long-term needs, challenges and cross-national demands was incorporated into the continuing development process of the SRIA. This was followed by the application of a top-down approach, analyzing at a macro-level all important European policies and strategic planning documents, action plans or consultation documents (communications, white papers and foresight reports) for topics impacting the agrifood sector or related to metrological issues linked to food, nutrition or public health (12). Key documents consulted (Figure 1) were the FAO Agrifood Challenges (13), UN Sustainable Development Goals (14), ESFRI Road Maps 2018 (5) & 2021 (3), Food 2030 Plovdiv Declaration (15), European Bioeconomy Strategy (16), Horizon Europe Missions (17–19), One Health European Joint Programme (20), EU Green Deal (21) & Farm to Fork Strategy (22), Food Safety Regulatory Research Needs 2030 (EFSA Strategy 2020) (23), UN 2030 Agenda (14), and impacts of the COVID-19 pandemic on food systems (24, 25). All challenges and topics touched upon therein that were deemed relevant to the METROFOOD-RI field of action or, in the wider sense, to society in the context of the major challenges or pressing issues of our time, were extracted, complemented with the results of the bottom-up approach, and then clustered into 8 dimensions of societal challenges whose thematic areas were largely derived from the 17 Sustainable Development Goals and targets set by the United Nations in *The 2030 Agenda for Sustainable Development* (14). The compiled topic collection was evaluated by the METROFOOD-RI consortium partners and expanded to include missing subjects identified as being important by the highly qualified experts within the team.

### 2.2. Locating key SRIA priorities via survey

Thematic prioritization was accomplished through an online vote by the Management Committee (MC) of the RI, consisting of ten voting members. The consultation was conducted using a customized in-house developed questionnaire divided into four blocks covering pre-defined topics related to the METROFOOD-RI operational components of “Metro” side, “Food” side, Electronic-RI (e-RI) and Producer/consumer interface and communication (Supplementary Figure S1). As part of the physical RI, the “Metro” side refers to analytical facilities devoted to the development of analytical methods and devices and the development and production of reference materials, while the “Food” side embraces experimental fields/farms/fisheries and food production/storage/preparation facilities and plants. The e-RI primarily relates to electronic and digital resources provided by the infrastructure. Based on the collected topics, the Producer/consumer affairs and external communications component emerged as a thematic co-category and was integrated into the survey as a separate discipline.

MC member were asked to answer the following questions: (Q1) “*Which of these topics do you consider most important for the scientific & research orientation of METROFOOD-RI?*,” and (Q2) “*From your point of view, which of these topics addresses an existing gap and should therefore be prioritized?*.” For the e-RI, only the second question was posed. Regarding the producer/consumer interface, MC members were asked “*On which of given topics/activities METROFOOD-RI should particularly focus on?*.” The survey included a 10-point numerical response scale ranging from zero points (no importance at all) to 10 points (extremely important), with boxes to be checked accordingly, as well as a “no answer” (NA) option to avoid arbitrary responses. An overall score was calculated for each topic within a block based on the survey voting results. For missing information in the questionnaire where NA was ticked, the median imputation technique (26) was applied to mitigate non-response bias. In cases where two questions on identical topics were asked within a survey block, such as for the “Metro” and “Food” side, separate subtotals were calculated for each question, and then summed up from both questions. This resulted in a maximum score of 200 for two, and 100 for a single survey question. A self-determined cut-off scoring system was introduced to classify the topics in the survey blocks as high, medium, or low priority following the same procedure in all the four blocks. After sorting topics in descending order according to their score, the top 15% of topics within each block were classified as high-priority (total score of 200–170 points for two questions or 100–85 points for single questions). For medium, low, and very low priority topics, the gradations were adjusted evenly in 5% increments to ensure a harmonized approach and an appropriate number or high-priority subjects within each thematic block.

## 3. Results

**Figure 2:**
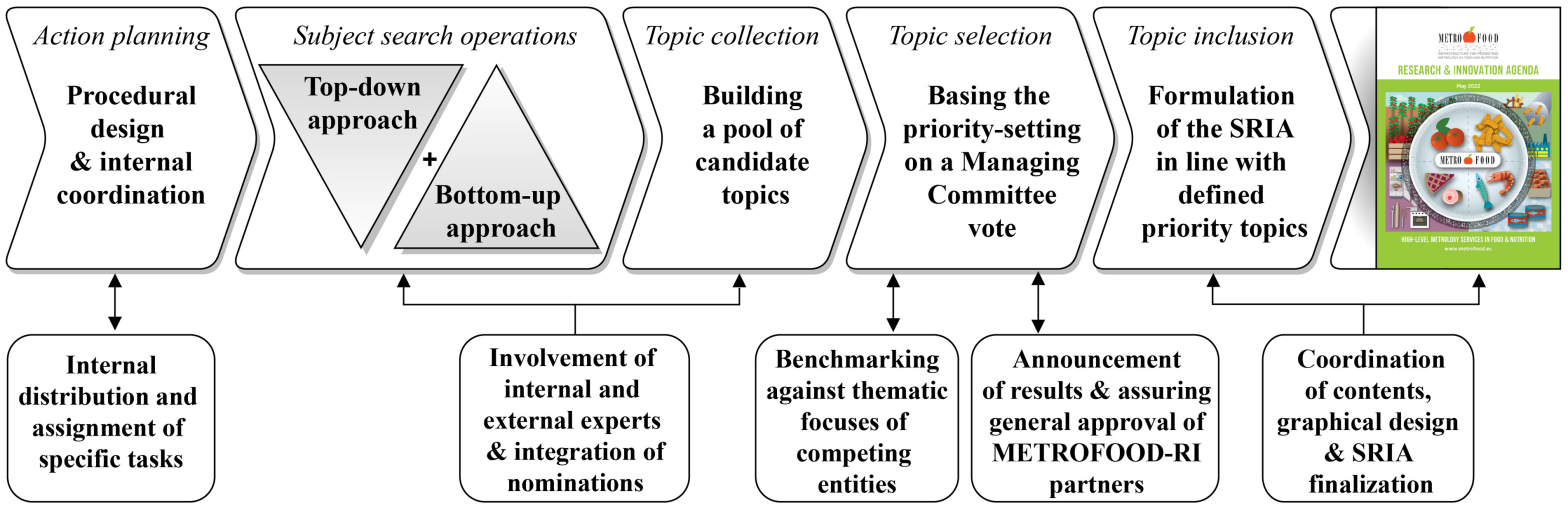
Basic workflow in building the SRIA of METROFOOD-RI.

**Figure 3:**
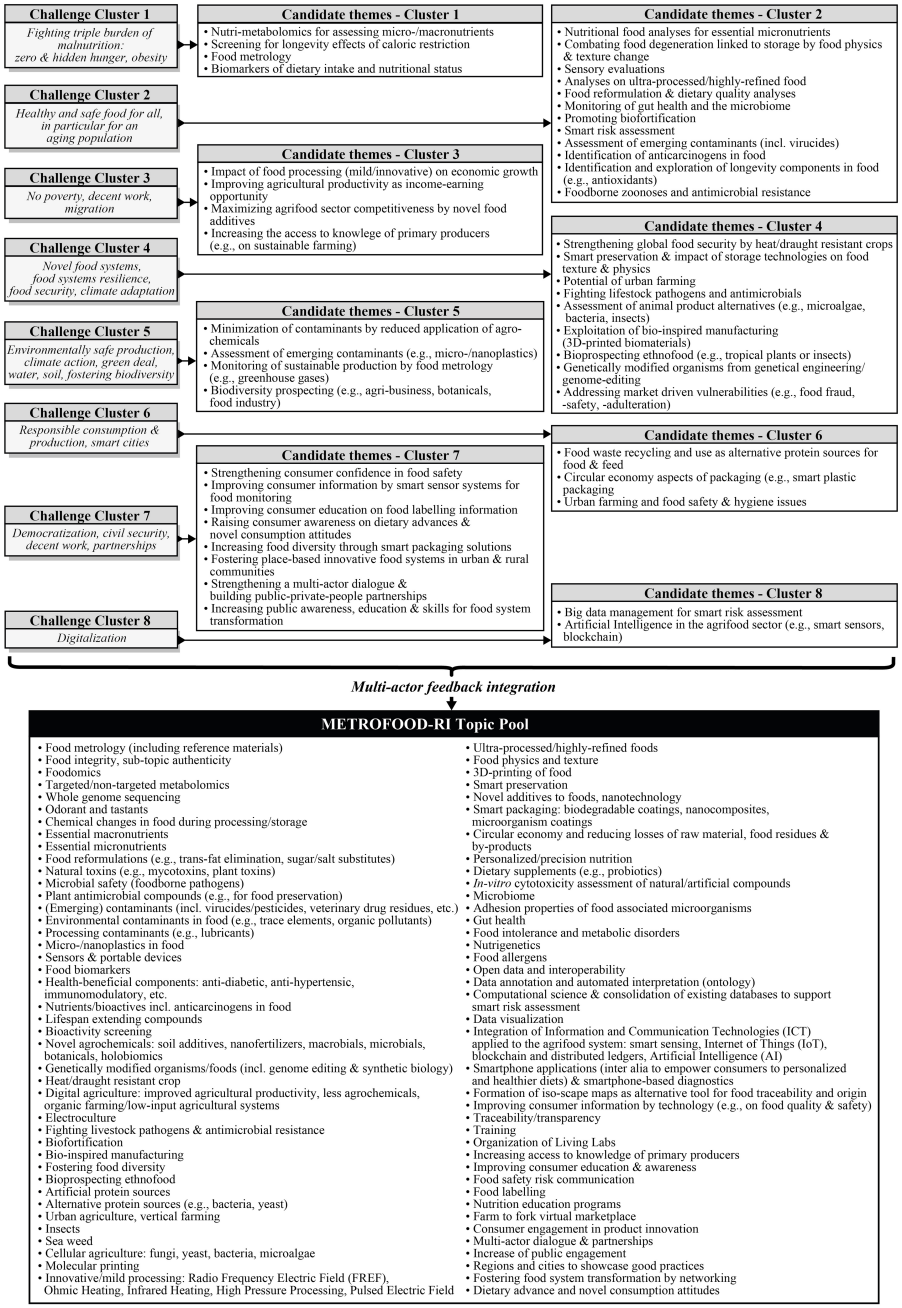
SRIA theme development process and candidate topic pool.

The combined approach of bottom-up and top-down, and active involvement and discussion with METROFOOD-RI partners and external stakeholders (Figure 2), identified 80 candidate topics as potential targets for the SRIA (Figure 3). The collected topic pool, largely based and derived from eight localized clusters of biggest global, societal and research related challenges, required further narrowing down to a few priority topics intended to serve as guiding themes for the SRIA. The subsequent prioritization through polling resulted in nine topics of high priority, 16 topics of medium priority, and 19 topics of low and 36 of very low priority over all thematic blocks. Only the high and medium priority topics provided the contextual framework for the METROFOOD-RI SRIA and are addressed in greater detail within this section (Table 1).

**Table 1:** Prioritization framework model for topics categorized as high and medium priority.

METROFOOD-RI component	Associated topics	Q1. Perceived importance (sum score of max. 100; *n*_Q1_ = 10)	Q2. Classification as priority/perceived gap-filling potential (sum score of max. 100; *n*_Q2_ = 10)	Total score (points) Q1. + Q2.	NA-responses for Q1. and/or Q2. (in %)^c^	Priority ranking
“Metro” side^a^	Food metrology (incl. reference materials)	95 (SD = 0.71)	86 (SD = 1.07)	181 (SD = 1.00)	0	High
	Food integrity, sub-topic authenticity	88 (SD = 1.03)	82 (SD = 1.32)	170 (SD = 1.19)	0	High
	(Emerging) contaminants	86 (SD = 1.26)	84 (SD = 1.35)	170 (SD = 1.28)	5	High
	Micro-/nanoplastics in food	83 (SD = 1.49)	83 (SD = 1.42)	166 (SD = 1.42)	5	Medium
	Natural toxins (e.g., mycotoxins, plant toxins)	87 (SD = 1.16)	78 (SD = 1.55)	165 (SD = 1.41)	5	Medium
	Food biomarkers	81 (SD = 1.29)	84 (SD = 0.84)	165 (SD = 1.07)	5	Medium
	Foodomics	82 (SD = 1.32)	82 (SD = 1.62)	164 (SD = 1.44)	15	Medium
	Nutrients/bioactives incl. anticarcinogens in food	81 (SD = 1.91)	81 (SD = 1.60)	162 (SD = 1.71)	5	Medium
	Sensors & portable devices	74 (SD = 2.46)	86 (SD = 0.84)	160 (SD = 1.89)	5	Medium
“Food” side^a^	Circular bioeconomy & reducing losses of raw material, food residues & by-products of food production	89 (SD = 0.99)	89 (SD = 0.99)	178 (SD = 0.79)	5	High
	Alternative protein sources	85 (SD = 1.85)	82 (SD = 1.23)	167 (SD = 1.39)	5	Medium
	Food allergens	79 (SD = 1.20)	83 (SD = 0.95)	162 (SD = 1.07)	5	Medium
	Personalized/precision nutrition	79 (SD = 1.52)	82 (SD = 1.40)	161 (SD = 1.43)	5	Medium
e-RI^b^	Open data & interoperability	90 (SD = 1.33)	NA^d^	90 (SD = 1.33)	0	High
	Integration of ICT applied to the agrifood: smart sensing, IoT, blockchain, AI, distributed ledgers	89 (SD = 0.57)	NA	89 (SD = 0.57)	10	High
	Computational science & consolidation of existing databases to support risk assessment	85 (SD = 0.85)	NA	85 (SD = 0.85)	10	High
	Smartphone applications (i.e. to empower consumers to personalized and healthier nutrition) & smartphone-based diagnostics	83 (SD = 0.82)	NA	83 (SD = 0.82)	0	Medium
Producer/consumer interface and communication^b^	Improving consumer information by technology	NA	82 (SD = 0.63)	82 (SD = 0.63)	10	High
	Traceability/transparency	NA	90 (SD = 0.94)	90 (SD = 0.94)	0	High
	Improving consumer education & awareness	NA	84 (SD = 0.84)	84 (SD = 0.84)	10	Medium
	Fostering food system transformation by networking	NA	84 (SD = 1.35)	84 (SD = 1.35)	10	Medium
	Training	NA	83 (SD = 1.14)	83 (SD = 1.14)	20	Medium
	Food safety risk communication	NA	83 (SD = 1.34)	83 (SD = 1.34)	10	Medium
	Food labelling	NA	81 (SD = 1.20)	81 (SD = 1.20)	10	Medium
	Dietary advances & novel consumption attitudes	NA	81 (SD = 2.08)	81 (SD = 2.08)	0	Medium

a Cut-off scores ranging from 200–0 points, with total score ratings between 200–170 points classified as high priority and <170–160 points as medium priority.

b Cut-off scores ranging from 100–0 points, with total score ratings between 100–85 points classified as high priority and <85–80 points as medium priority.

c Total number of respondents to question Q1: *n*_Q1_ = 10; to question Q2: *n*_Q2_ = 10; to Q1 + Q2: *n*_Q1 + Q2_ = 20.

d NA, Not applicable.

With an overall score ranging between 181–170 points, food metrology (180 points), food integrity & authenticity and (emerging) contaminants (170 points each) were identified as priority areas for action on the “Metro” side. Medium priority was allocated to the subjects of micro-/nanoplastics in food (166 points), natural toxins (e.g., mycotoxins, plant toxins) (165 points), food biomarkers (165 points), Foodomics (164 points), nutrients/bioactives including anticarcinogens in food (162 points), and sensors and portable devices (160 points). Regarding the “Food” side, highest priority was assigned to the wider topic of circular economy and associated activities of reducing losses of raw materials, food residues and by-products of food production, with a total of 178 points. Medium-action priority was allocated to the topics of alternative protein sources (167 points), food allergens (162 points) and personalized/precision nutrition (161 points). Moreover, as far as it concerned the e-RI, high-priority was placed on open data and interoperability (90 points), integration of Information and Communication Technologies (ICT) applied to the agrifood (*inter alia* smart sensing, Internet of Things (IoT), blockchain and distributed ledgers, Artificial Intelligence (AI)) (89 points), and computational science and consolidation of existing databases to support risk assessment (85 points). Medium-priority was recorded for smartphone applications along with smartphone-based diagnostics (83 points). At the interface between producers and consumers, first priority was assigned to the task of improving consumer information by technology (e.g., on food quality and safety) (92 points), and the traceability/transparency within the agrifood sector (90 points). Ranging between 84–81 points, medium-priority was assigned to the improvement of consumer education and awareness (84 points), fostering food system transformation by networking (84 points), training (83 points), food safety risk communication (83 points), food labelling (81 points), and dietary advances and novel consumption attitudes (81 points).

## 4. Discussion

As a strategic framework, a SRIA communicates the general, specific and operational goals and the corresponding target action areas of science-oriented entities or research infrastructure for a defined period of time, most commonly the next 5–10 years. In many cases, there is little transparency about how the thematic areas are established in different SRIAs, or what techniques were applied for topic identification and prioritization. Hence, this work aims at presenting in a transparent mode the workflows preceding the launch of the METROFOOD-RI SRIA in order to serve as a source of inspiration and guidance for other research networks or businesses. In this regard, this discussion refrains from elaborating on the identified priority topics of the METROFOOD-RI SRIA in a corresponding context of action and their relevance, as this can be read in detail in the released final version of the SRIA publicly accessible (26). In this view, the narrative presented herein centers on sharing and outlining in more detail the challenges and issues faced in identifying METROFOOD-RI strategic action areas, and what has been done to address them.

In order to confine the focal topics of a SRIA, it is imperative to conclude on a comprehensive understanding of the existing and emerging issues that are directly or indirectly relevant to a project or undertaking, such as a research infrastructure. For this reason, within METROFOOD-RI, an inventory analysis of most important strategic and policy documents was conducted. It was, however, inevitable to concentrate on a pool of selected documents that had been published within a certain period of time in order to advance the work on finalizing the SRIA within a given timeframe. It necessarily follows that theme-based additions from successor publications, such as for instance beyond the European Food Safety Authority (EFSA) Strategy 2027 (27) or some further Horizon Europe work programmes with extensions to the current active programme of 2021/2022 (17), could certainly have revealed new relevant topics of interest or attention that might have gone unnoticed due to an established internal METROFOOD-RI cut-off date. The original collection of topics could therefore not claim to be all-encompassing. The second step, consequently, required additional consultation with experts within the METROFOOD-RI consortium, who provided nuanced feedback based in part on the engagement of other professionals in their own networks and communication to external stakeholders from the political, scientific, industrial, civil society, and academic communities.

The 48 partners of the METROFOOD-PP consortium from 18 countries, organized as National Node networks, and operating in diverse areas of research and development, consumer sciences, and information and communication technologies, performed as a kind of “internal think tank” of great leverage, giving a very inclusive collection of topics coarsely tailored to the vision and missions of METROFOOD-RI.

A far greater challenge than the topic identification was the selection of a suitable approach for further prioritization. The original intention was to develop a set of transparent and self-defined criteria on which to base the topic prioritization for the SRIA. In research and strategic planning, it is a widely used practice to set priorities based on predefined decision criteria (28, 29). The criteria were to be derived from a large-scale inventory project conducted within METROFOOD-PP that aimed at mapping the expertise, capacities, (technical) resources, and service offerings of all contributing partners for a total of 285 facilities (“Metro” side: 144 facilities; “Food” side: 51 facilities; e-RI: 90 facilities) (30, 31), and a separately performed survey on subjective views and personal perceptions of intragroup professionals. The following are examples of criteria that were originally intended as a baseline: (1) number of existing and active facilities in individual subject areas; (2) level of competence and expertise per subject area and/or individual topics; (3) service offerings that are already installed and operational, respectively offerings available after minor upgrades; (4) individual thematic preferences in terms of the topics collection for the SRIA; (5) perceived and existing research gaps in individual scientific disciplines; (6) pressing topics with a high urgency for action; (7) existence of the necessary in-house resources and infrastructure for the practical implementation of research activities on individual subject areas. The plan was not only to set priorities based on a multi-criteria catalog and an applied numeric scoring system, but also to assign a weighting factor to each criterion, giving more weight to particularly important criteria. The practical implementation of the prioritization task revealed several problems, which in the end did not allow for a fully satisfactory solution. The main challenge was to capture the multitude of METROFOOD-RI partners and their various action spectra in the different research areas in such detail and completeness, prior to transfer into a suitable scoring system, to allow the prioritization approach to be based on valid underpinning data. This also involved mapping of mutual networking possibilities in terms of current knowledge base and future acquisition, technical abilities and future service offering capabilities of participating laboratories and processing units. To put this in more concrete terms, there were, for example, topics that were identified in the previous topic collection pool as potential targets for METROFOOD-RI activities, but where none of the partners was actively conducting research up to that time (e.g., 3D-printing of food, artificial protein sources, personalized nutrition). In this context, it was a matter of argument whether, for example, one should award zero points with regard to the first prioritization criterion mentioned above, meaning that there is no RI unit working in this specific field, while for another topic, say in the metrology subject, up to 130 active laboratories have been spotted. As a result, individual topics could have been deemed less relevant due to a lower overall score achieved and dropped from the METROFOOD-RI priority list owing to their sub-representation, even though their societal or policy relevance might have been significant. This may be also due to the fact that at the time of preparing the SRIA, METROFOOD-RI was still in its preparatory phase and covered only a limited number of fully-ready or fully-integrable resources and capabilities, which are expected to expand continuously to reach operational maturity.

Even though the inventory of METROFOOD-RI facilities could not be used for priority setting, it provided very valuable information for the management and future planning of the RI, as it gave a good impression on existing competences, gaps and deficits in specific thematic areas and shed light on where its strengths and highest performance potentials lie. In selecting the future core topics for the METROFOOD-RI SRIA, it was considered important to have broad agreement from the majority of the consortium in order to maintain and strengthen a sense of community and cohesion in the longer term. However, discussions on the best approach to setting priorities within the RI revealed that opinions and views on the prioritization of individual topics vary widely, and are sometimes strongly influenced by one’s own research areas, competencies, and interests.

In provision of a representative decision and in order to drive the SRIA preparation forward in time, the METROFOOD-RI MC vote on this matter was taken into consideration, as described in greater detail in the previous methods section. Though a vote among ten MC members does not ensure necessarily a representative majority of the entire RI of 48 partners, this compromise was not rejected by anyone. Basing the voting on a limited eligible sample was also an acceptable trade-off, as the areas of expertise and research fields of each MC member were quite heterogeneous and covered a large spectrum of topics related to the physical and electronic RI components. Moreover, as all MC members were concurrently work package leaders in the METROFOOD-PP project and involved in the landscape analysis and the overall user mapping, the voting provided a very representative cross-section among all the RI facilities. Given that assessments on the relevance and priority of individual topics tended to be subjective in nature, a subjective bias, among other things, based on the knowledge background of the survey respondents, cannot be completely ruled out. This was however tried to be reduced to a minimum by integrating a “no answer” response option in the questionnaire, if no particular competence or knowledge existed on certain topics, which was to be expected with regard to the wide array of thematic areas. Due to the large number of topics identified as possible flagship topics for the SRIA, it was inevitable to prioritize and, thus, limit and define the content framework. The determination of a proper cut-off score on the basis of which a ranking of topics by priority was possible, depended heavily on the outcomes of the survey, but was intended to be as transparent, plain, and comprehensible as possible. A different sorting of the threshold values could might have resulted in significantly more or fewer priority topics, taking into account that the total scores obtained for individual topics were in part quite close to each other. The decision on the scoring intervals was made solely on a pragmatic consideration to ultimately arrive at a manageable number of core subjects for the METROFOOD-RI SRIA.

Having identified the METROFOOD-RI SRIA priority topics, it was equally important to contrast them against the research portfolio of other (research) organizations within and outside Europe in order to explore competitive advantages and strategically expand the RI through increased collaborative efforts in SRIA-related activities. Last but not least, it was carefully considered how to sustainably strengthen the market position of the RI and promote innovation potentials in a targeted, collaborative and effective manner.

All in all, a SRIA should not be viewed as a static but rather a living document and roadmap with a validity horizon of several years, as in the case of METROFOOD-RI for the initial period of 5 years (2021–2025). Once the SRIA of METROFOOD-RI was established (32), further planning of its update process was also initiated in order to maintain an up-to-date status in the next years and to drive strategic alignment with critical research needs and emerging challenges, as well as new opportunities and market-driven (technical) developments and upcoming trends. For the time being, it remains to be seen whether further step-by-step optimization is necessary to maximize the efficiency and effectiveness of the RI in its daily work under the legal status of an ERIC, thus contributing significantly to the promotion and exploitation of European research potential and to the strengthening of the European Research Area at large.

## 5. Conclusion

This report described the methodology and main outcomes of the research study that helped to identify the key thematic areas for the SRIA of METROFOOD-RI from a metrological standpoint, to articulate critical and emerging issues and demands and to structure how the integrated facilities of the RI can operate in the first 5 years of operation as an ERIC. Setting-up a SRIA is often a tedious process that demands team efforts and professionalism, time and not least endurance. The present study illustrated that there is not one ultimate way or procedure to follow, often requiring a certain degree of flexibility in handling unanticipated challenges, pragmatism and spirit of compromise are decisive factors to succeed.

With a view to the METROFOOD-RI SRIA, from the project idea to its concretization and final implementation, a participatory development process was set in motion that incorporated the different opinions, views and positions of the various affiliated partners and stakeholders. Establishing and implementing a common research agenda for METROFOOD-RI, with a focus on metrology in food and nutrition, required a targeted and differentiated approach and a concentration on selected research and business areas deemed as highly important and relevant, also in view of the major societal challenges and the priority action areas delineated for the RI.

After having acquired the relevant basics, the aim is now to take METROFOOD-RI to the next level and to turn its vision and mission into joint actions, guided by the SRIA objectives, and concrete results. In providing research communities with integrated cross-discipline services, knowledge and resources, METROFOOD-RI represents a valuable addition to Europe’s scientific community and the European research infrastructures landscape, standing in the starting gates to generate real impact at multiple levels at the scientific and technological and innovation, socio-economic, political, or environmental dimensions. By developing and maintaining strategic alliances with other research infrastructures and scientific networks, it is expected that the impact emanating from METROFOOD-RI will not only increase, but also multiply its potential to benefit society at large and as an important pillar of the European Research Area.

## Data availability statement

The original contributions presented in the study are included in the article/Supplementary material, further inquiries can be directed to the corresponding author.

## Ethics statement

Ethical review and approval was not required for the study on human participants in accordance with the local legislation and institutional requirements. Written informed consent for participation was not required for this study in accordance with the national legislation and the institutional requirements.

## Author contributions

TS and MR initiated and performed the research work, interpreted the data, and prepared the manuscript. MT, NB, and CZ have made a substantial, direct and intellectual contribution to the work. All authors contributed to the article and approved the submitted version.

## Funding

This research was funded by the METROFOOD-PP project (EU-Horizon 2020-INFRADEV-2018-2020/H2020-INFRADEV-2019-2, GA No. 871083).

## Conflict of interest

The authors declare that the research was conducted in the absence of any commercial or financial relationships that could be construed as a potential conflict of interest.

## Publisher’s note

All claims expressed in this article are solely those of the authors and do not necessarily represent those of their affiliated organizations, or those of the publisher, the editors and the reviewers. Any product that may be evaluated in this article, or claim that may be made by its manufacturer, is not guaranteed or endorsed by the publisher.

## Footnotes

^1^ www.metrofood.eu
